# ﻿Generic placement of the African flea beetle *Polycladamaculipennis* Bryant and the possible occurrence of the genus *Procalus* Clark in sub-Saharan Africa (Coleoptera, Chrysomelidae, Galerucinae, Alticini)

**DOI:** 10.3897/zookeys.1145.90667

**Published:** 2023-02-03

**Authors:** Paola D'Alessandro, Maurizio Biondi

**Affiliations:** 1 Department of Life, Health & Environmental Sciences, University of L’Aquila, Via Vetoio-Coppito, 67100 L’Aquila, Italy University of L'Aquila Coppito Italy

**Keywords:** Afrotropical region, Neotropical region, taxonomy, new combination, lectotype designation

## Abstract

*Polyclada* Chevrolat and *Procalus* Clark are flea beetle genera (Coleoptera, Chrysomelidae, Galerucinae, Alticini). *Polyclada* is endemic to the Afrotropical region, while *Procalus* has never been described outside of the Neotropical region. The new combination *Procalusmaculipennis* (Bryant, 1942), **comb. nov.** is proposed for *Polycladamaculipennis* Bryant, 1942. Its plausible type locality is Venezuela, and not Cameroon, as recorded on the labels of the type material, and hence the occurrence of *P.maculipennis* in Africa is questionable.

## ﻿Introduction

*Polyclada* Chevrolat, 1836 is an Afrotropical flea beetle genus occurring in sub-Saharan Africa, Saudi Arabia, and Yemen. It comprises 16 described species currently under revision (Biondi and D’Alessandro 2010, 2012; Biondi et al. 2022). The genus is associated with Anacardiaceae (*Sclerocaryabirrea* (A. Rich) Hochst.) and Burseraceae (*Commiphora* spp.), in a variety of woodland and savannah ecosystems (Chaboo et al. 2007; Iannella et al. 2021). *Polyclada* is one of the five Afrotropical genera belonging to the *Blepharida*-group *sensu*Furth and Lee (2000) and Prathapan and Chaboo (2011), along with *Diamphidia* Gerstaecker, *Xanthophysca* Fairmaire, and the recently re-evaluated *Blepharidina* Bechyné and *Calotheca* Heyden (Biondi et al. 2017, 2019; D’Alessandro et al. 2018, 2019, 2020, 2021). The *Blepharida*-group currently comprises 21 genera from the Afrotropical, Nearctic, Neotropical, and Oriental regions (Medvedev 1999; Furth and Lee 2000; Prathapan and Chaboo 2011; Biondi et al. 2017). Furth and Lee (2000) provided a morphological synthesis of the group based on adult characters (tarsal claws, procoxal cavities, head, pronotum, hind femora, eye, proepimeron, and metatibia) and larval characters (antenna, mandible, labrum, stemmata, endocarina, coronal suture, and frontal suture). However, some characters are shared by most (but not all) of the genera (Furth and Lee 2000), and a more comprehensive analysis based on the whole set of genera and representative species is badly needed to more rigorously test the monophyly of the group.

During revisionary studies of the genus *Polyclada*, we examined the type material of *P.maculipennis* Bryant, 1942, in the general collection of the Natural History Museum in London (NHMUK) and noticed that it belongs to the Neotropical genus *Procalus* Clark, 1865. Bryant (1942) based the description of this species on three specimens from Cameroon and believed it to be allied to *Polycladabohemani* (Baly, 1861).

*Procalus* comprises an unknown number of species, but including *P.mutans* (Blanchard, 1851), *P.viridis* (Philippi & Philippi, 1864), *P.lenzi* (Harold, 1876), *P.reduplicatus* Bechyné, 1951, *P.malaisei* Bechyné, 1951, and *P.silvai* Jerez, 1995 (Jerez 1992, 1995). Three more species were reported by Artigas and Solar (2015): *P.artigasi* Jerez, *P.ortizi* Jerez, and *P.vilosensis* Jerez, which are also cited by other authors (Jerez 2003; Prathapan and Chaboo 2011). However, they were described in an unpublished doctoral thesis (Jerez 1999a, cited by Jerez 2003), therefore, according to Article 9.12 of the International Code of Zoological Nomenclature (ICZN 2020), these three names cannot be considered as available.

In this paper, we revise the taxonomic status of *Polycladamaculipennis* and discuss the possible occurrence of the genus *Procalus* in sub-Saharan Africa.

## ﻿Materials and methods

Examined material consisted of dried, pinned specimens preserved in the institutions listed below. The specimens were examined and dissected under a Leica M205C stereomicroscope. Photographs were taken using a Leica DMC5400 camera and were compiled using Zerene Stacker v. 1.04. Scanning electron micrographs were taken using a Hitachi TM-1000. Abbreviations of the depositories follow Evenhuis (2022).

### ﻿Depositories

**BAQ** collection of M. Biondi, University of L’Aquila, Italy;

**MSNG**Museo Civico di Storia Naturale “Giacomo Doria”, Genova, Italy;

**NHMB**Naturhistorisches Museum, Basel, Switzerland;

**NHMUK**The Natural History Museum, London, United Kingdom;

**NMPC**National Museum (Natural History), Prague, Czech Republic.

## ﻿Results

### ﻿ Procalus maculipennis

Taxon classificationAnimaliaColeopteraChrysomelidae

(Bryant, 1942) comb. nov.

421B5B24-5870-506F-9784-8FB162B43101

Figs 1–3 , 5–8

Polyclada maculipennis Bryant, 1942: 164.

#### Type material examined.

**Figures 1–4.**
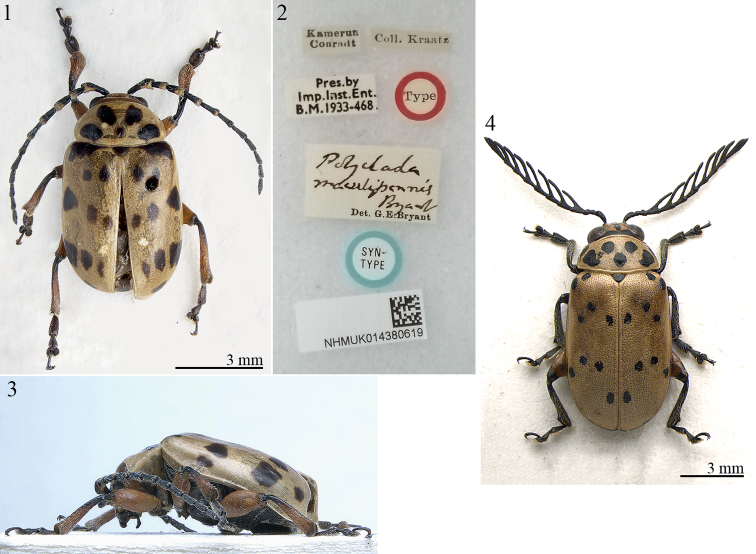
Lectotype of *Polycladamaculipennis* Bryant, 1942 **1** habitus, dorsal view **2** ibid., labels **3** ibid., habitus, lateral view **4***Polycladabohemani* Baly, male, Kenya (BAQ), habitus.

Lectotype of *Polycladamaculipennis* ♂: “Kamerun Conradt // Coll. Kraatz // Pres. By Imp. Inst. Ent. B.N. 1933-468 // *Polycladamaculipennis* Bryant / Det. G.E. Bryant” (NHMUK) (here designated by M. Biondi and P. D’Alessandro) (Figs 1–3). Paralectotypes of *Polycladamaculipennis*, 2♀♀: same data as for lectotype (NHMUK).

#### Material of *Procalus* species examined for comparison.

Chile: *Procalusreduplicatus*, syntype, 1♀, “Chile // Coll. Nickerl / Mus. Pragense // *Procalusmutans* Blchd. // Typus // *Procalusreduplicatus* n. sp. Type ♀ / 1948 Det. J. Bechyně” (NMPC); Procaluscf.viridis, 1 specimen, El Tabo, Valparaiso, 29 Sept. 1986 (NHMB); Procaluscf.lenzi, 1 specimen, Santiago, Pudahuel, 20 Nov. 1986 (NHMB); Procaluscf.malaisei, 2 specimens, D. Ed. Varas Arangua leg., 1921 (MSNG); Procaluscf.lenzi, 1 specimen, ibid (MSNG); Procaluscf.viridis, 1 specimen, Viña del Mar, Valparaiso, May 1899, F. Silvestri leg. (MSNG); Procaluscf.silvai, 1 specimen, Concepcion, 1903, P. Herbst leg. (MSNG); Procaluscf.reduplicatus, 1 specimen, ibid (MSNG); Procaluscf.mutans, 1 specimen, Concepcion, Sept. 1903, P. Herbst leg. (MSNG); *Procalus* sp., 2 specimens, ibid (MSNG); Procaluscf.reduplicatus, 1 specimen, Concepcion, 1904, P. Herbst leg. (MSNG); *Procalus* sp., 1 specimen, ibid (MSNG).

#### Remarks.

*Polyclada* can be immediately distinguished from similar genera by the antennae, longer than half body length, with antennomeres 4–10 pectinate or flabellate in male and serrate in female (Fig. 4). Characters of the antenna along with other morphological features, such as procoxal cavities open posteriorly, antennomere 4 at least double length of antennomere 3, as well as elytral punctation always confused, densely and uniformly impressed, permits identification of the genus within the *Blepharida* group in the Afrotropical region (Biondi and D’Alessandro 2012; Biondi et al. 2017).

**Figures 5–8.**
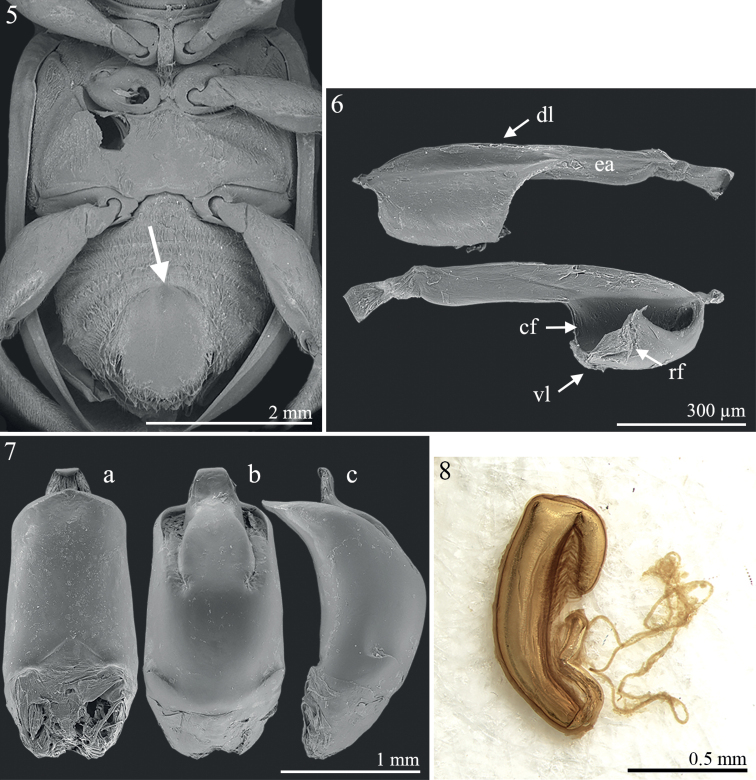
*Procalusmaculipennis* Bryant, 1942 comb. nov. **5** ventral parts in male **6** metafemoral extensor tendon **7** median lobe of aedeagus in ventral, dorsal, and lateral views (**a–c**, respectively) **8** spermatheca. Abbreviations: cf: central furrow; dl: dorsal lobe; ea: extended arm; rf: recurved flange; vl: ventral lobe.

The type material of *Polycladamaculipennis* has all the diagnostic characters of *Procalus* (Clark 1865; Jerez 1992), none of which occur in any *Polyclada* species: antennae with antennomeres 1–5 flattened compared to 6–11, especially in male, and antennomere 1 clearly distally enlarged, and strikingly serrated in male (Fig. 1; Clark 1865; Jerez 1992: figs 4A, 5A, 6A, 7A, 8A, 1995: figs 1, 2); fifth abdominal sternite in male with a wide, deep, oval depression (Fig. 5; Jerez 1992: figs 5E, 6D, 7C, 8F, 1995: fig. 2); metafemoral extensor tendon simplified, very slender (Fig. 6; Furth and Suzuki 1994: fig. 6b; Jerez 1992: figs 2, 3D): dorsal lobe straight, with very elongate extended arm; central furrow very wide; ventral lobe subtriangular; recurved flange short, poorly sclerotized. Additionally, the median lobe of aedeagus and spermatheca (Figs 7, 8) are typical of the *Procalus* species (median lobe of aedeagus: Furth and Suzuki 1994: fig. 6a; Jerez 1992: figs 4E, 5C, 6E, 7B, 8G, 1995: fig. 10; spermatheca: Furth and Suzuki 1994: fig. 6c; Jerez 1992: figs 4D, 5D, 6B, 7D, 8E, 1995: fig. 11). Median lobe of the aedeagus of the lectotype here designated (Fig. 7): thickset and smooth; in ventral view tapering towards the apex, and slightly narrowing subapically; apex subtriangular, widely obtuse, with a small median tooth; in lateral view, clearly curved. Spermatheca of the paralectotypes (Fig. 8): subcylindrical and elongate basally, curved towards the ductus attachment; distal part distinctly bent and about 1/3 the basal part in length; ductus basally inserted, short, uncoiled, moderately thickset. We therefore propose the new combination *Procalusmaculipennis* (Bryant, 1942), comb. nov.

## ﻿Discussion

Clark (1865) reported the genus *Procalus* as abundant and widely distributed in Chile, and also present in Brazil and Bolivia. Based on Scherer (1983), this genus occurs in Chile, Bolivia, Brazil, and Argentina. Jerez (1992, 1995) considered *Procalus* as occurring only in Chile, based on her examined material. Later, she (Jerez 1999b) stated that, based on both public and personal collections, the genus is distributed between latitudes of 30°50'S and 40°50'S, and cited undetermined *Procalus* material from southern Argentina preserved in the Museum National d’Histoire Naturelle de Paris. Endemic to Chile or not, it has never been described outside of the Neotropical region. What about *Procalusmaculipennis* comb. nov. from Cameroon? We can only speculate about some different hypotheses. Hypothesis A: the genus *Procalus* has a Gondwanan distribution, even though it is dramatically more abundant in the Neotropical region. The Gondwanan distributions of terrestrial taxa generally refer to genera with clearly differentiated species, or higher taxa (cf. Gómez-Zurita and Cardoso 2021). Among flea beetles, the genera with Afrotropical–Neotropical disjunct distributions occur in the two regions with clearly differentiated species: *Terpnochlorus* Fairmaire from the Afrotropical region, Venezuela, and Mexico, and, if the synonymy is confirmed, the Malagasy *Abrarius* Fairmaire, is possibly a senior synonym of *Gioia* Bechyné from South America (Biondi and D’Alessandro 2012). A second Afrotropical–Neotropical disjunct distribution is observed at a higher taxonomic level; for example, *Zomba* Bryant is the only representative of the subtribe Monoplatina in the Afrotropical region. This subtribe occurs almost exclusively in the Neotropical and southern part of the Nearctic regions and is present in the Australian region only with the genus *Opisthopygme* Blackburn, 1896. Based on the diagnostic characters reported by Jerez (1992), *Procalusmaculipennis* comb. nov. is so similar to *P.reduplicatus* Bechyné that one could evaluate to establish a synonymy in a possible future revision of the genus. For Hypothesis A to be true, these two taxa would have remained so similar despite approximately 135 million years of independent evolution (cf. Donateli Gatti et al. 2021), even though the diversification of *Procalus* in South America occurred in a much shorter time, likely during the Pliocene (Jerez 1999b). Hypothesis B: the species was imported to Africa via host plants. *Procalus* species are associated with *Lithraea* Miers ex Hook. & Arn. and *Schinus* L. (Anacardiaceae) (Jerez 1992, 1999b). The genus *Lithraea*, native to South America, is reported as introduced only in California and Tunisia, not in Cameroon or other sub-Saharan countries (POWO 2021). *Schinus* is also native to South America, is used ornamentally around the world, and was imported into several African countries, but not into Cameroon and adjacent areas (POWO 2021).

Hypothesis C: the types are mislabelled. Starting from the assumption that Leopold Conradt was the collector, it is possible that the entomological material he collected or somehow acquired in Venezuela (where he stayed for some time before 1889) was brought to Cameroon, where he subsequently collected in 1896 (Rohlfien 1975). In fact, material from Venezuela reached the Deutschen Entomologischen Instituts—now Senckenberg Deutsches Entomologisches Institut (SDEI)—via Gustav Kraatz in 1905 along with material from Togo and Cameroon (Rohlfien 1975). It is plausible that the samples from Venezuela were mixed up with, and then erroneously labelled as being from Cameroon. A similar interpretation about the putative and unlikely disjunct distributions of some Hymenoptera species have been offered by Liston et al. (2017). Similarly, Furth (1998) highlighted that *Blepharidasemisulcata* Achard originally described from Cayenne (French Guiana) is a mislabelled specimen from the Afrotropical Region. For us, this is the most plausible hypothesis to explain the alleged presence of *Procalus* in Africa. However, fieldwork in Cameroon and Venezuela in search of the original habitat and host plants of *Procalusmaculipennis* comb. nov. can put the issue to rest.

## Supplementary Material

XML Treatment for Procalus maculipennis
